# Supplementary material for: Short-Lived Antibody-Mediated Saliva Immunity against SARS-CoV-2 after Vaccination
Source: Microbiol Spectr. 2023 Mar 6;11(2):e04947-22. doi: 10.1128/spectrum.04947-22 (PMC10101069; doi:10.1128/spectrum.04947-22)
Supplement: Supplemental file 1 — Supplemental material. Download spectrum.04947-22-s0001.pdf, PDF file, 0.04 MB [file spectrum.04947-22-s0001.pdf]

# Short-lived antibody-mediated saliva immunity against SARS-CoV-2 after vaccination

## Supplemental information:

### Table of contents:

**Supplementary Figures:** 2

**Supplementary Figure 1.** Validation tests of antibodies in saliva against RBD from SARS-CoV-2 in the Luminex assay 2

**Supplementary Figures:**

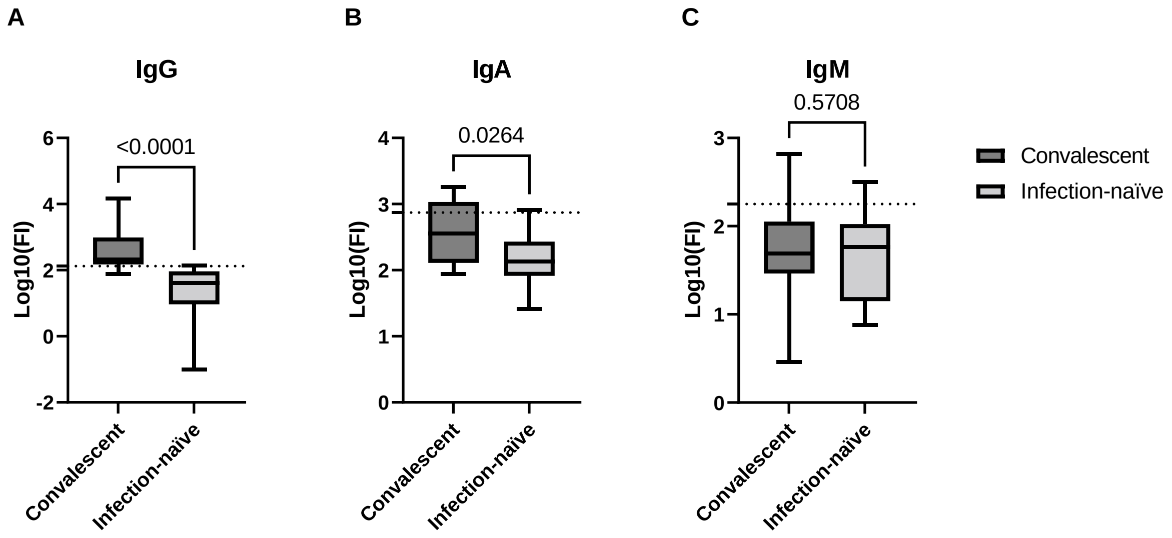


**Supplementary Figure 1. Validation tests of antibodies in saliva against RBD from SARS-CoV-2 in the Luminex assay.** Boxplots showing the level of antibodies in Log10(Fluorescence Intensity [FI]) in convalescent individuals and infection-naïve individuals. SARS-CoV-2 infection was confirmed by a positive RT-PCR result. All samples were collected before vaccination. Levels of IgG antibodies (A), levels of IgA antibodies (B), and levels of IgM antibodies (C) in convalescent and infection-naïve individuals. The horizontal dotted line indicates the assay cutoff. The p-values have been calculated using a Mann-Whitney test. $p<0.05$ was considered significant.
